# Detection of 4a,5-dihydropravastatin as Impurity in the Cholesterol Lowering Drug Pravastatin

**DOI:** 10.3390/molecules26154685

**Published:** 2021-08-03

**Authors:** Wibo B. van Scheppingen, Peter P. Lankhorst, Marcus Hans, Marco A. van den Berg

**Affiliations:** DSM Biotechnology Center, 2613 AX Delft, The Netherlands; peter.lankhorst@dsm.com (P.P.L.); marcus.hans@dsm.com (M.H.); marco.berg-van-den@dsm.com (M.A.v.d.B.)

**Keywords:** dihydrostatins, biosynthesis, finished dosage forms, active pharmaceutical ingredient, mass spectrometry, NMR

## Abstract

Dihydro analogues are known byproducts of the fermentative production of statins and cannot be detected with existing pharmacopoeia analysis methods. We detected dihydropravastatin in most commercial formulations of pravastatin with LC-MS, in some cases in levels requiring identification. In fermentation broth samples of the single step production of pravastatin, we detected and identified for the first time 4a,5-dihydropravastatin, and confirmed that after several recrystallization steps this impurity can be fully removed from the pravastatin powder.

## 1. Introduction

**Figure 1:**
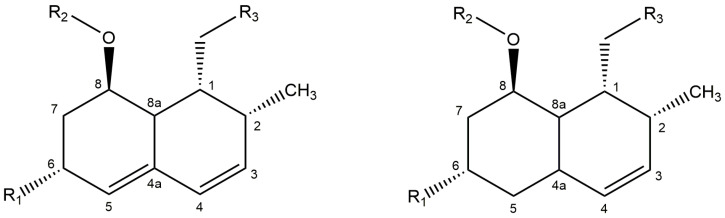
General structure and atom numbering of main fermentation derived statins. Left: Compactin (R1 = –H, R2 = –OCOCH(CH_3_)CH_2_CH_3_), Pravastatin (R1 = –OH, R2 = –OCOCH(CH_3_)CH_2_CH_3_), Lovastatin (R1 = –CH_3_, R2 = –OCOCH(CH_3_)CH_2_CH_3_), Simvastatin (R1 = –CH_3_, R2 = –OCOC(CH_3_CH_3_)CH_2_CH_3_), for all R3 = –CH_2_CH_2_CHOHCH_2_CHOHCH_2_COOH. Right: dihydro core structure.

Statins are a class of widely prescribed pharmaceuticals, mimicking the substrate of the key enzyme of human cholesterol biosynthesis, (3S)-hydroxy-3-methylglutaryl-coenzyme A (HMG-CoA) reductase. Through competitive inhibition it effectively causes a reduction in plasma cholesterol levels, thereby preventing hypercholesterolemia. The first generation of statins–compactin (syn. ML-236B, mevastatin), lovastatin (syn. monacolin K, mevinolin), pravastatin (syn. eptastatin) and simvastatin–are structurally related (Figure 1) and either isolated or derived from fungi [1,2,3]. The second (e.g., atorvastatin) and third generations (e.g., rosuvastatin) are structurally more diverse synthetics with improved physicochemical properties and reduced IC50 (concentrations required for 50% inhibition) [4]. For the analysis of pravastatin and impurities in finished dosage forms such as prescribed tablets, official pharmacopoeia monographs are available (like USP and Ph. Eur.). After sample preparation the analysis is performed on an HPLC with separation on a reversed phase C18 column and UV-detection. The compounds are detected at the maximum absorption wavelength of pravastatin at 238 nm. The amount of pravastatin is determined by comparison with a standard solution with a known concentration of pravastatin 1,1,3,3-tetramethylbutylamine salt. Any impurities in the samples are identified based on the relative retention times as compared to pravastatin. The area percent of the peaks in the UV chromatogram at 238 nm are used for the quantification of these impurities.

Regulations require that impurities higher than 0.05% are reported, higher than 0.1% are identified, and higher than 0.15% are qualified. Dihydrostatins are lacking one of the two conjugated double bonds typical for the statin core structure (Figure 1) and have therefore no UV absorption at the 238 nm wavelength used in the pharmacopeia methods. In the literature several methods have been described for the analysis of the “dihydro” derivatives of statins. After derivatization of dihydrolovastatin and dihydrocompactin to their 4-nitrobenzoate derivates, these compounds can be analysed with HPLC-UV at 260 nm [5]. Albers-Schönberg et al. [6] determined dihydrolovastatin after BSTFA (N,O-Bis(trimethylsilyl)trifluoroacetamide) derivatization with GC/MS. ^1^H-NMR at 600 MHz allowed for the simultaneous detection of (0.01%) dihydrolovastatin in lovastatin [7]. Several authors have described the analysis of these compounds with (UP)LC using a mass spectrometer to detect the protonated molecular ion and the fragmentation pattern of dihydrosimvastatin [8,9].

**Figure 2:**
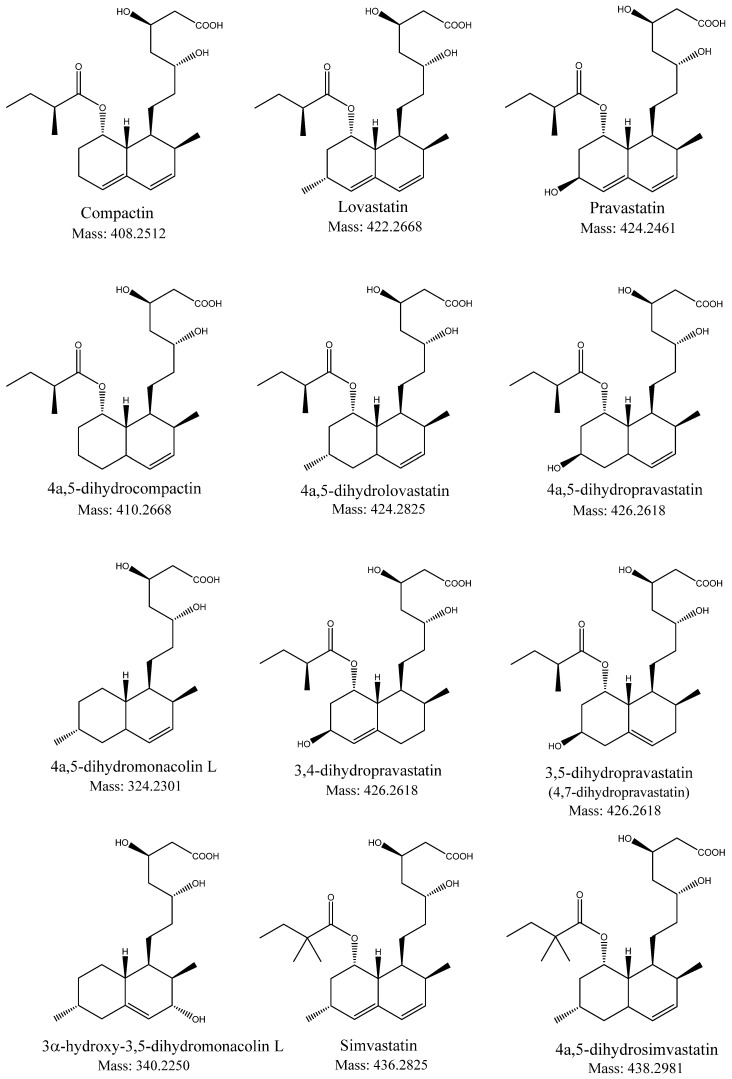
Structures of relevant statins and dihydrostatins.

Surprisingly, little information is known in the literature about the occurrence of dihydropravastatin. Li et al. [10] isolated dihydropravastatin after bioconversion, and elucidated the structure by means of X-ray crystallography and NMR. The same compound was reported earlier as part of a synthetic drug optimization program, and a full set of NMR data and assignments was given [11]. Interestingly, both groups identified the impurity as 3,5-dihydropravastatin (The original paper [10], using a different nomenclature, reports 4,7-dihydropravastatin. Here, we follow the same carbon numbering (Figure 1) but use the generally accepted nomenclature [12]), with the remaining double bond in the C4-C4a position, while later 3,4-dihydropravastatin with the remaining double bond in the C4a-C5 position was also reported in pravastatin sodium preparations from several manufacturers [13]. This is in contrast to all of the other, above mentioned “dihydrostatins”, which are of the 4a,5-type (having the remaining double bond in the C3-C4 position (Figure 1 and Figure 2)). Here, we describe for the first time the detection of 4a,5-dihydropravastatin in the fermentation broth of the single-step fermentation process of pravastatin [14].

## 2. Results

### 2.1. LC-MS Analysis of Dihydropravastatin during Pravastatin Fermentation

During the development of the one-step fermentative production of pravastatin in *Penicillium chrysogenum* [14] several peaks were detected with MS that were not visible in the standard UV-chromatogram as recorded at 238 nm (Supplementary Figure S1). This was a strong indication for the absence of one or both conjugated double bonds in the decalin ring system, initiating the monitoring for dihydropravastatin presence at all steps in the process: fermentation, downstream processing, and the pravastatin sodium end product.

In fermentation broth one compound gave a mass spectrum comparable to compactin but with a 2 Da higher molecular weight (data not shown). The lack of UV absorption at 238 nm and the higher mass were indications for a known byproduct during fermentation of compactin, namely: 4a,5-dihydrocompactin (Figure 2), previously identified based on ^1^H-NMR and accurate mass measurements [15].

**Figure 3:**
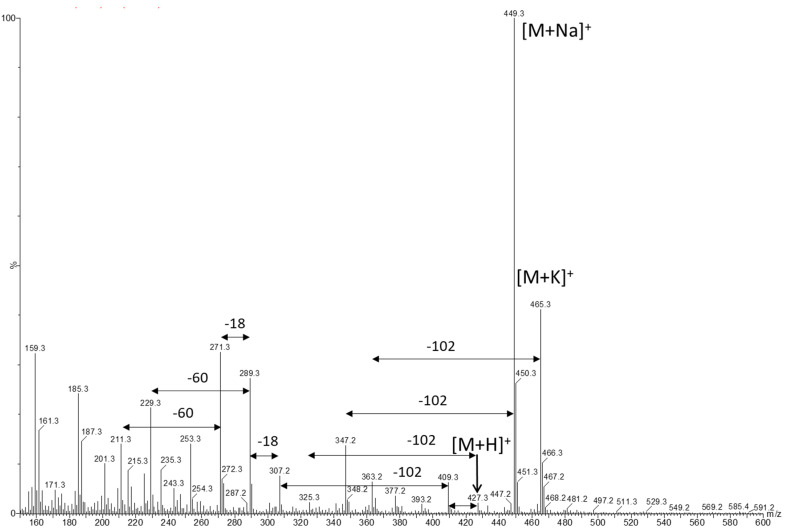
Positive ion MS spectrum of dihydropravastatin.

Another peak resembled the MS spectrum of pravastatin but, again, with a 2 Da higher molecular ion peak with several adducts ([M + H]^+^, [M + Na]^+^ and [M + K]^+^). The MS fragmentation spectrum (Figure 3) showed losses typical for statins as already described in the literature: an α-methylbutyrate group (C_5_H_10_O_2_, −102) from the ester side chain, an acetate (C_2_H_4_O_2_, −60) from the ẟ-lactone and loss of water (H_2_O, −18) [1,9,13]. As for all the mass fragments a difference of 2 Dalton was observed as compared with pravastatin, this proves that the mass difference is located in the backbone of the molecule with the hexahydronaphthalene ring system. Accurate mass measurement on an LTQ orbitrap gave an m/z of 449.25097 for the [M + Na]^+^ and the corresponding molecular formula of C_23_H_38_O_7_Na. Compared with the theoretical mass of the sodium adduct of dihydropravastatin, there is a difference of only −0.010 ppm.

### 2.2. Detection of Dihydropravastatin Impurity in Pravastatin Formulations

**Figure 4:**
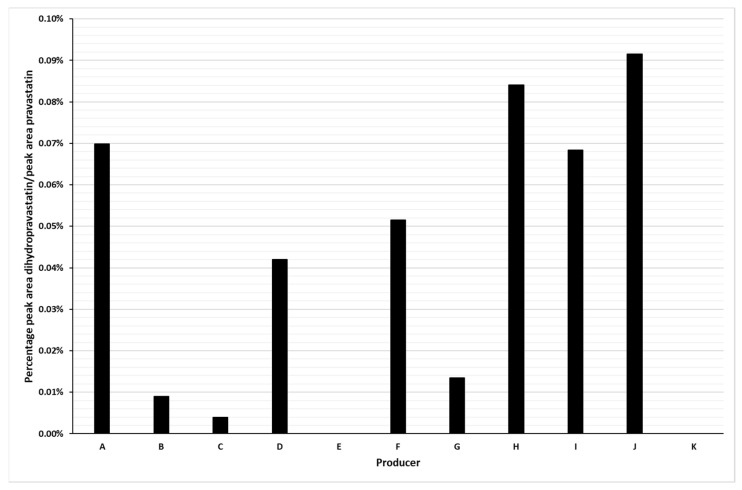
Relative levels of dihydropravastatin in commercial samples (finished dosages, A–I; API, J), and API from single step fermentation (K) after subsequent recrystallization according to [16]. Percentage of peak area dihydropravastatin relative to peak area pravastatin.

As previously described [7] dihydro impurities are difficult to remove from fermentation broths during downstream processing (Supplementary Figure S2). A recrystallisation step via diamine salts proved to be an effective method for the removal of all impurities from pravastatin in process samples as obtained in the single step fermentation, including the dihydropravastatin derivative [16]. This allowed for the production of an active pharmaceutical ingredient (API) of pravastatin sodium salt without a detectable amount of dihydropravastatin (Figure 4, sample K; Supplementary Figure S3). As a comparison we also analyzed several commercially available pravastatin finished dosage forms (Figure 4, samples A–I) and an alternative commercial API (Figure 4, sample J) for the presence of dihydropravastatin. In eight of the nine tablets and in the API dihydropravastatin was detected. A proper quantification of the dihydropravastatin with LC-MS is not possible without an isotope-labelled internal standard and a reference compound with known purity, therefore we quantified the peak area of dihydropravastatin relative to the peak area of pravastatin. In a recent paper, levels of the 3,4-dihydropravastatin isomer were quantified in ten pravastatin samples from different manufacturers with UPLC-UV/MS, ranging from 0.01–0.10% [13] (similar levels as we observed in our study).

### 2.3. Identification of 4a,5-dihydropravastatin

**Figure 5:**
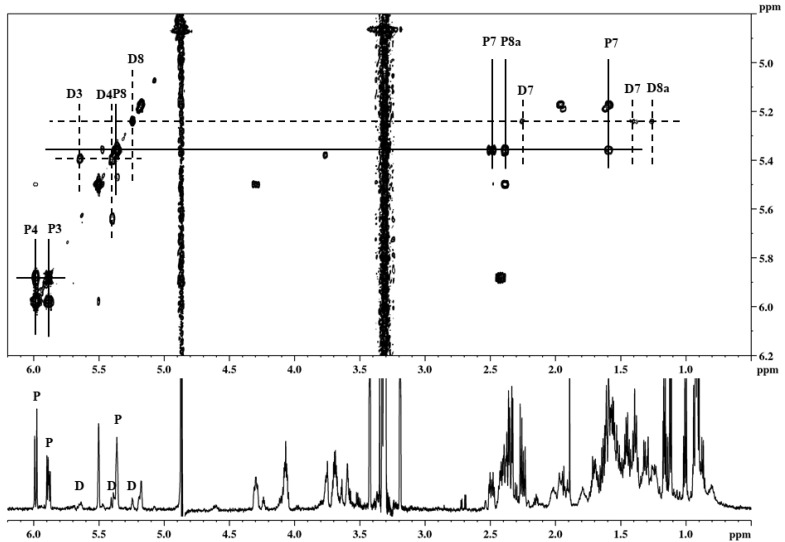
NMR spectra (COSY and 1H) of HPLC fraction obtained from mother liquor. Signals marked “P” are assigned to pravastatin, signals marked “D” are assigned to 4a,5-dihydropravastatin.

Due to the low concentration of dihydropravastatin in pravastatin samples, it was difficult to obtain a fraction of dihydropravastatin of relatively high purity for identification purposes. Therefore, a small sample originating from a purification step was prepared, containing pravastatin and 4a,5-dihydropravastatin as an enriched impurity. NMR analysis of this fraction confirmed that the isolated fraction contains pravastatin and the impurity in a ratio of approximately 100:15 (Figure 5). From the NMR spectra, it was possible to conclude unequivocally that the major impurity in this fraction was indeed 4a,5-dihydropravastatin since, by means of a COSY experiment, the two coupled pairs of protons H3–H4 and H8–H8a were easily detected. The intensities in the TOCSY spectrum are lower, and therefore in the TOCSY spectrum only H3 and H4 of the dihydropravastatin were detected. H3 appears as a characteristic doublet with a coupling constant J = 9.8 Hz, due to coupling with H4. This coupling constant is identical to the one observed in pravastatin, and indicates the presence of a double bond with two cis protons attached. The chemical shifts of H3, H4 and, most importantly, H8a in 4a,5-dihydropravastatin differ in a very characteristic manner from the analogous chemical shifts of pravastatin. This is illustrated by the chemical shifts of lovastatin, compactin, simvastatin, and monacolin L and their 4a,5-dihydro analogues taken from literature and recorded in this study (Table 1). Δδ values are defined as δ(parent compound)–δ(4a,5-dihydro analogue), and the Δδ values of the above-mentioned statins are compared with the same values found for pravastatin and the isolated impurity. H8a always shifts approximately 1.1 ppm upfield when the double bond between 4a and 5 is replaced by a single bond. H8 shifts 0.2 ppm, H4 0.6 ppm, and H3 0.1 ppm, all in the upfield direction. In addition, the positions of both H7 protons of the impurity were observed in the COSY spectrum. It is clear from Figure 5, that both H7 protons have shifted approximately 0.2 ppm upfield with respect to the positions of those protons in pravastatin. A similar upfield shift can be found when the 4a,5-dihydro analogues of simvastatin and lovastatin are compared with the parent compounds. It should be noted that the chemical shifts of both H7 protons of pravastatin are significantly different from those of the other statins mentioned, due to the influence of the OH-group on C6.

These data strongly supported that the isolated impurity must be 4a,5-dihydropravastatin. In particular, the shift towards higher field of H8a of approximately 1.1 ppm is very indicative of the absence of the 4a,5 double bond. For the sake of comparison, the chemical shifts and Δδ values are also given of the isomer (3,5-dihydropravastatin) with a double bond between carbon atoms C4 and C4a (Table 1). It is obvious, that the chemical shift of H3 in this compound differs strongly from the chemical shift of H3 in 4a,5-dihydropravastatin, as well as the Δδ of H8a.

NMR data of the synthetic lactone of 4a,5-dihydropravastatin confirmed the chemical shifts observed by us. Although the data are presented without assignment of the signals, it is clear from the presence of a signal at 5.64 ppm (H3, tentative assignment by the authors of this paper) that, indeed, the remaining double bond must be in the 3,4 position [17]. DeCamp et al. [18] synthesized the 3,5-dihydro analogue of lovastatin and, in this case, indeed, the absence of a signal of H3 in the double bond region of the NMR spectrum confirmed that in a 3,5-isomer, the remaining double bond must be in the 4a,4 position. In the course of this study pravastatin sodium salt from Sigma Aldrich was investigated by NMR, and we found that this contains the same 4a,5-dihydropravastatin impurity as the fermentation broth samples (Supplementary Figures S4 and S5). The resolution and sensitivity were sufficient to confirm the identity and assign many of the ^1^H and ^13^C signals without prior purification. The concentration of 4a,5-dihydropravastatin in this product was almost 2% with respect to pravastatin (data not shown). The important cross-peaks of the impurity showed connections in the TOCSY spectrum of both H3 and H4 with the methyl group on C2, confirming the location of the double bond in the decalin ring. Furthermore, both H4 and H6 correlated with H4a, and the position of the two H5 protons was detected. An HSQC spectrum and a zoom into the crowded high-field region of the HSQC spectrum confirmed the identity of the impurity: the olefinic proton/carbon pair H5/C5 of pravastatin is missing, and, instead a CH2 appears at app 41 ppm, and an additional signal assigned to H4a/C4a appears at 31 ppm, and the strong upfield shift of H8a is clearly visible (Supplementary Figures S6 and S7). The assigned signals are listed in Table S1 of the Supplementary Material.

**Table 1:** NMR chemical proton shifts of statins.

Compound	H3		H4		H8		H8a		Ref.
	ppm	Δδ	ppm	Δδ	ppm	Δδ	ppm	Δδ	
Monacolin L	5.72	0.13	5.91	0.61	(a)	(a)	(a)	(a)	[19]
4a,5 dihydromonacolin L (Δ3,4)	5.59		5.30		(a)		(a)		
Compactin	5.71	0.11	5.95	0.52	5.33	0.13	-	-	[1,15]
4a,5 dihydrocompactin (Δ3,4)	5.60		5.43		5.20		1.22		
Lovastatin	5.78	0.13	6.00	0.62	5.40	0.19	2.26	1.07	[6,9]
4a,5 dihydrolovastatin (Δ3,4)	5.65		5.38		5.21		1.19		
Lovastatin	5.78	0.14	5.99	0.60	5.38	0.20	2.26	1.07	[7]
4a,5 dihydrolovastatin (Δ3,4)	5.64		5.39		5.18		1.19		
Lovastatin	5.84	0.15	6.01	0.59	5.35	0.20	2.37	-	[20] (b)
4a,5 dihydrolovastatin (Δ3,4)	5.69		5.42		5.15		-		
Lovastatin	-	-	-	-	-	-	-	-	[18] (d)
3,5 dihydrolovastatin (Δ4,4a)	-		5.48		5.28		-		
Simvastatin	5.78	0.13	5.99	0.60	5.37	0.18	2.26	1.06	This study
4a,5 dihydrosimvastatin (Δ3,4)	5.65		5.39		5.19		1.20		
Pravastatin	5.88	3.80	5.99	0.45	5.40	0.04	2.32	0.50	[11]
3,5 Dihydro pravastatin (Δ4,4a)	2.08		5.54		5.36		1.82		
Pravastatin	-	-	-	-	-	-	-	-	[10]
3,5 Dihydro pravastatin (Δ4,4a)	2.06		5.51		5.33		-		
Pravastatin	-	-	-	-	-	-	-	-	[17] (d)
4a,5 dihydropravastatin lacton (Δ3,4)	5.64		5.41		5.30		-		
Pravastatin	5.91	0.23	6.00	0.59	5.38	0.13	2.38	1.09	This study (c)
4a,5 dihydropravastatin (Δ3,4)	5.68		5.41		5.25		1.27		

(a) H8 and H8a chemical shifts not published and due to different substitution at C8 a comparison is not meaningful; (b) solvent is CD_3_CN:D_2_O 80:20; (c) solvent is CD_3_OD; (d) assignment not given in reference. Tentative assignment by the authors of this paper.

## 3. Discussion

### 3.1. Biosynthetic Pathway

In all the dihydro derivatives of which the structure is elucidated by NMR and other spectroscopic techniques the saturation is at the 4a,5 bond (4a,5-dihydrolovastatin, 4a,5-dihydromonacolin L, 4a,5-dihydrocompactin [7,15,19] and the pharmacopoeia reference standard 4a,5-dihydrosimvastatin (Table 1)). Only for pravastatin a structure previously was reported in literature identified as 3,5-dihydropravastatin [10,11]. Amongst all-natural statins, the biosynthetic pathway for lovastatin has been studied most extensively [9,12,21]. Biosynthesis starts with an iterative polyketide synthase (LNKS, lovastatin nonaketide synthase) generating the lovastatin backbone, 4a,5-dihydromonacolin L acid. As known from other polyketides, subsequent transformation steps (often alkylations and/or oxidations) convert it into the final product. Here, a second polyketide synthase (LDKS, lovastatin diketide synthase) synthesizes the 2-methylbutyryl side chain (on the R2 position, Figure 1), which is attached to the core. A key reaction in completing the conjugated ring structure is the hydroxylation and subsequent dehydration by a cytochrome P450 monooxygenase to create monacolin L and monacolin J [12]. However, this reaction can be insufficient and, in that case, the remaining 4a,5-dihydromonacolin L and/or 4a,5-dihydromonacolin J are likely to be decorated with the 2-methylbutyryl side chain, yielding 4a,5-dihydrolovastatin [6,22].

Similarly, the biosynthesis of pravastatin also requires two analogous polyketide synthases, as well as a P450 monooxygenase, to form the compactin intermediate [23]. Subsequently, pravastatin is produced by enzymatic hydroxylation of compactin by a bacterial P450 [14], which most likely at the same time also will hydroxylate the 4a,5-dihydrocompactin present in the fermentation broth [15] into 4a,5-dihydropravastatin. Promiscuity of statin P450 enzymes with respect to the absence or presence of both double bonds in the decalin core or the methylbutyrate side chain was demonstrated by C6 hydroxylation of dihydromonacolin L [22] and lovastatin [24], making it very likely that 4a,5-dihydropravastatin is derived from hydroxylating 4a,5-dihydrocompactin by the P450 monooxygenase.

The previously reported isomer 3,5-dihydropravastatin [10,11] could originate from double bond migration during the hydroxylation of 4a,5-dihydrocompactin. In the biosynthesis of lovastatin, 3α-hydroxy-3,5-dihydromonacolin L, was shown to be an intermediate during the first oxidation, although unstable [12]. From a synthetic chemical perspective such a double bond migration is not illogical because a double bond on a more substituted carbon is known to be more stable. In the octalin structure the ring conformation and steric effects of the side groups may also play an important role. During the synthesis of dihydrolovastatin by selective reduction of lovastatin, formation of a product with a rearrangement of the double bond position to give the 3,5-dihydrolovastatin was observed [18]. Moreover, an active inhibitor of HMG-CoA reductase isolated from *Aspergillus sclerotiorum* [25], confirming that stable 3,5-dihydro variants of statins do exist.

### 3.2. Biological Activity of Dihydrostatins

Statins have four structural features relevant for their biological activity: the δ-lactone moiety (R3, Figure 1), the ester side-chain (R2, Figure 1), the make-up of the decalin ring and the substituents on the decalin ring (e.g., R1, Figure 1). The presence of a δ-lactone moiety, being the element analogous to the natural substrate HMG-CoA, is essential for its activity, while modifications often lead to activity reduction [26]. Absence of the ester side-chain reduces activity [2], while modifications of and on the decalin ring gave ambiguous results. Whereas all the reported 4a,5-dihydro statins (4a,5-dihydrolovastatin; 4a,5-dihydrocompactin; 4a,5-dihydromonacolin L; and 4a,5-dihydropravastatin) are potent inhibitors of HMG-CoA reductase [6,15,17,19], 3,5-dihydropravastatin showed no inhibitory effect on HMG-CoA reductase [10], suggesting that the position of the double bond might be relevant for activity. However, other statins with a C4-C4a double bond, (e.g., 3,5-dihydroxy-3,5-dehydrolovastatin, 3′α-iso-pravastatin (obtained after rearrangement on the decalin ring) and iso-simvastatin-6-one) are reported being medium to highly active [25,27,28]. Moreover, a variant with a fully saturated decalin ring, 4,5,6,7-tetrahydropravastatin, was similarly effective as pravastatin [29], confirming that modifications such as the hydrogenation of one or more of the double bonds in the decalin ring system are not destroying the activity of the statin, but merely modifying the effectiveness.

## 4. Materials and Methods

### 4.1. Chemicals and Reagents

For the analysis of the finished dosage forms, a pH 5.6 buffer solution was prepared with 0.1 M sodium acetate (ACS Reag. Ph Eur, Merck, Darmstadt, Germany) adjusted to pH with acetic acid (glacial 100%, ACS.ISO reag. Ph Eur, Merck, Darmstadt, Germany). Methanol (LiChrosolv^®^, Merck, Darmstadt, Germany) was used as solvent and eluent for the LC separations. Triethylamine (Sigma-Aldrich, Taufkirchen, Germany) and formic acid (98–100%, ACS Reag. Ph Eur, Merck, Darmstadt, Germany) were added to the mobile phases for the LC analysis. Water was prepared in house with a Milli-Q system (Millipore, Bedford, MA, USA). Acetonitrile (LiChrosolv^®^, Merck, Darmstadt, Germany) was used as mobile phase for the LC analysis.

Pravastatin 1,1,3,3-tetramethyl amine (European Pharmacopoeia reference standard catalogue code Y0000201, batch 2) was used as a reference compound. Pravastatin sodium salt hydrate for NMR studies was purchased from Sigma-Aldrich (Germany, P4498).

Simvastatin (Ph. Eur. Reference standard CRS) and dihydrosimvastatin (impurity K, Ph. Eur. Reference standard CRS) were obtained from the European Directorate for the Quality of Medicines & Health Care (edqm).

Deuterated methanol (Cambridge Isotope Laboratories, Andover, MA, USA) was used as a solvent for the NMR analysis.

### 4.2. LC-MS Analysis

The pravastatin finished dosage forms were analyzed according to the USP method. Six tablets were dissolved in 500 mL acetate buffer at pH = 5.6 and diluted five times with a mixture of buffer and methanol (80:20). These samples were analyzed with HPLC-UV and high resolution (Orbitrap) MS.

Fermentation samples were extracted with acetonitrile/water (1:1) and analysed after centrifugation with LC-UV/MS. Pravastatin salts, as acquired after downstream processing (DSP) procedure, were dissolved in acetonitrile/water (50:50) and injected.

An Acquity UPLC equipped with a Photodiode Array (PDA) detector (Waters, Milford, MA, USA) and Quattro Micro triple quadrupole (tandem) MS (Micromass, Manchester, UK) equipped with an electrospray probe in the positive mode (ESI) and controlled by Waters MassLynx software were used for analysis. The separation was performed at 40 °C on a Waters Sunfire C18 column with the dimensions 150 × 4.6 mm and a particle size of 3.5 µm (Waters, Wexford, Ireland), with the following settings: a split ratio of 700 µL to the PDA and 500 µL to the MS, a flow rate of 1.2 mL/min, solvent A (60% methanol + 39.9% Milli-Q water + 0.1% formic acid) and B (acetonitrile with 0.04% formic acid), 0–5 min 100% A, 5–15 min 40% A and 60% B, 16–17 min isocratic

The PDA range was 190–400 nm (resolution 1.2 nm) with a sampling rate of 10 Hz. The MS was performed in ES+ scan mode with mass range *m*/*z* 150–600, a scan duration of 0.8 s, and an interscan delay of 0.05 s.

The high-resolution mass spectrometer system consisted of an Accela LC pump, Accela PDA detector (at 238 nm) and an LTQ orbitrap with electrospray probe (Thermo Scientific GmbH, Bremen, Germany). The LTQ orbitrap was operated in the positive mode and the data recorded as profile scan in the mass range *m*/*z* 150–1000 and at a resolution of 60,000. The separation was performed at ambient temperature on a Chromolith column Speed ROD RP-18e with the dimensions of 4.6 × 50 mm. The eluent compositions were methanol/water/acetic acid/triethylamine (TEA) 500:500:1:1. The flow rate was 1.00 mL/min that was split after the column with 200 µL/min to the MS and 800 µL/min to the UV-detector. The system was operated by the Thermo Xcalibur™ mass spectrometry data system (Thermo Scientific, Bremen, Germany).

### 4.3. NMR Analysis

A process sample enriched in 4a,5-dihydropravastatin was collected from a mother liquor acquired during the downstream processing of the fermentation broth [16]. This sample was extracted with acetonitrile/water (1:1) and purified by preparative liquid chromatography. The extract was injected on an Alliance 2790 HPLC (Waters, Milford, MA, USA) with a ZQ 2000 single quadrupole MS (Micromass, Manchester, UK) equipped with an electrospray probe in the positive mode. The system was controlled by Waters MassLynx software. The separation was performed on a Waters XTerra prep MS C18 column with the dimensions 7.8 × 100 mm and a particle size of 3.5 µm (Waters, Wexford, Ireland). The fraction containing the dihydropravastatin was collected manually based on the MS signal. The combined fractions were lyophilized and dissolved in CD_3_OD. NMR spectra of this sample enriched in dihydropravastatin were recorded on a Bruker Avance 600 equipped with a 5 mm TXI cryo-probe.

NMR spectra of 4a,5-dihydrolovastatin, simvastatin and 4a,5-dihydrosimvastatin were recorded on a Bruker Ascend 700 NMR spectrometer, equipped with a 5 mm cryo TCI probe. The assignment of signals was obtained by means of COSY and TOCSY spectra that were recorded with standard Bruker pulse programs cosygpprqf and mlevphpr. Spectra were recorded at 300 K, both with NS = 8 and 2K datapoints in the F2 dimension and 700 (COSY) or 512 (TOCSY) data points in the F1 dimension.

## 5. Conclusions

The presence of dihydro statins was first reported by researchers from Merck Sharp & Dohme in 1981 [5,6,15]. They discovered dihydrocompactin and dihydrolovastatin in fermentation samples of compactin and lovastatin, respectively. Later, the dihydro derivatives of precursors of statins and semi-synthetic statins, such as monacolin L and simvastatin were discovered as well [19,30]. Despite the fact that the dihydro impurities are present in all natural and semi-synthetic statins, not much has been published about these compounds. While statins are commonly used medicines, the analysis methods of the official monographs do not allow for the detection of dihydro statins. Moreover, dihydrosimvastatin is the only available dihydro reference compound (as impurity K) supplied by the European Pharmacopoeia.

Here, we reported for the first time the presence of 4a,5-dihydropravastatin in fermentation broth and confirmed the structure by NMR. The origin of the molecule likely resides in insufficient activity and/or selectivity of the P450 enzyme during the biosynthesis of compactin, the precursor for pravastatin. The subsequent hydroxylation step [14] will likely also accept 4a,5-dihydrocompactin as a substrate to form 4a,5-dihydropravastatin. Dihydro impurities were detected in most commercial pravastatin samples at levels up to about 0.1%. The provided UPLC-MS method offers a reliable detection of dihydropravastatin in process samples, API, and finished dosages.

## Data Availability

The data presented in this study are openly available for all figures and samples.

## Supplementary Materials

The following are available online. Supplementary Figure S1: LC-UV/MS chromatograms of a pravastatin fermentation sample with UV at 238 nm (top) and the MS total ion chromatogram (bottom); Supplementary Figure S2: LC-MS selected ion chromatogram at m/z 465 of a partially purified pravastatin sample with pravastatin detected as the potassium adduct of the (M + 2) isotope [M + 2 + K]^+^ and dihydropravastatin as [M + K]^+^; Supplementary Figure S3: LC-MS selected ion chromatogram at m/z 465 of the pravastatin API purified after recrystallization, with pravastatin detected as the potassium adduct of the (M + 2) isotope [M + 2 + K]^+^ and no dihydropravastatin; Supplementary Figure S4: COSY of pravastatin sodium salt (Sigma Aldrich) with connectivities of the 4a,5-dihydropravastatin impurity indicated with dashed lines; Supplementary Figure S5: TOCSY of pravastatin sodium salt (Sigma Aldrich) with connectivities of the 4a,5-dihydropravastatin impurity indicated with dashed lines; Supplementary Figure S6: HSQC of pravastatin sodium salt (Sigma Aldrich). Black numbers correspond to the assignment of pravastatin. The assignment of 4a,5-dihydropravastatin is indicated in blue, and the artefacts due to long-range couplings in pravastatin are indicated with red asterisks; Supplementary Figure S7: High-field region of the HSQC of pravastatin sodium salt (Sigma Aldrich). Black numbers correspond to the assignment of pravastatin. The assignment of 4a,5-dihydropravastatin is indicated in blue, and the artefacts due to long-range couplings in pravastatin are indicated with red asterisks; Supplementary Table S1: Chemical shifts of pravastatin and 4a,5 dihydropravastatin detected in methanol-d_4_ at 300 K. 5 mg of pravastatin from Sigma Aldrich. Chemical shifts taken from HSQC spectrum. Chemical shift reference: methanol (δ ^1^H = 3.300, δ ^13^C = 47.67).

## Sample Availability

Samples of the compounds are not available from the authors.
