# Supplementary material for: Detection of 4a,5-dihydropravastatin as Impurity in the Cholesterol Lowering Drug Pravastatin
Source: Molecules. 2021 Aug 3;26(15):4685. doi: 10.3390/molecules26154685 (PMC8347671; doi:10.3390/molecules26154685)

Detection of 4a,5-dihydropravastatin as impurity in the cholesterol lowering drug pravastatin
Wibo B. van Scheppingen, Peter P. Lankhorst, Marcus Hans and Marco A. van den Berg

Supplementary data

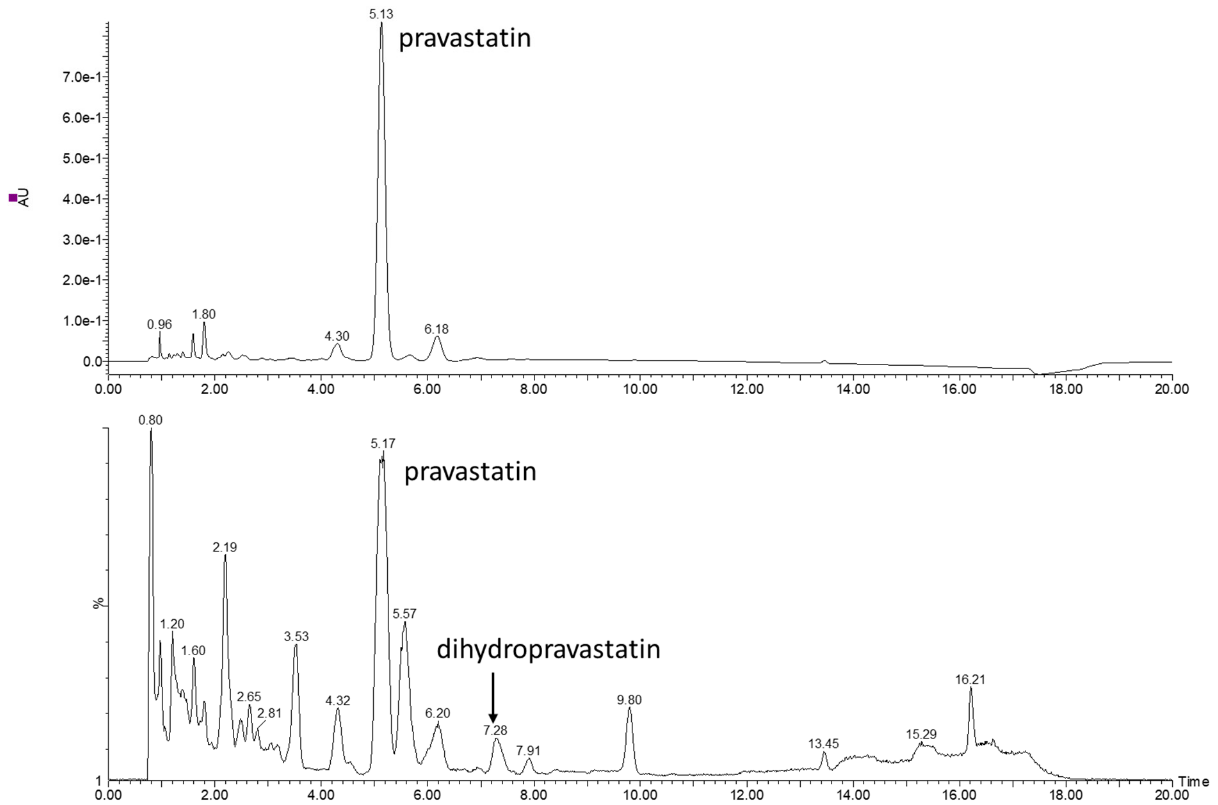

Supplementary Figure S1. LC-UV/MS chromatograms of a pravastatin fermentation sample with UV at 238 nm (top) and the MS total ion chromatogram (bottom)

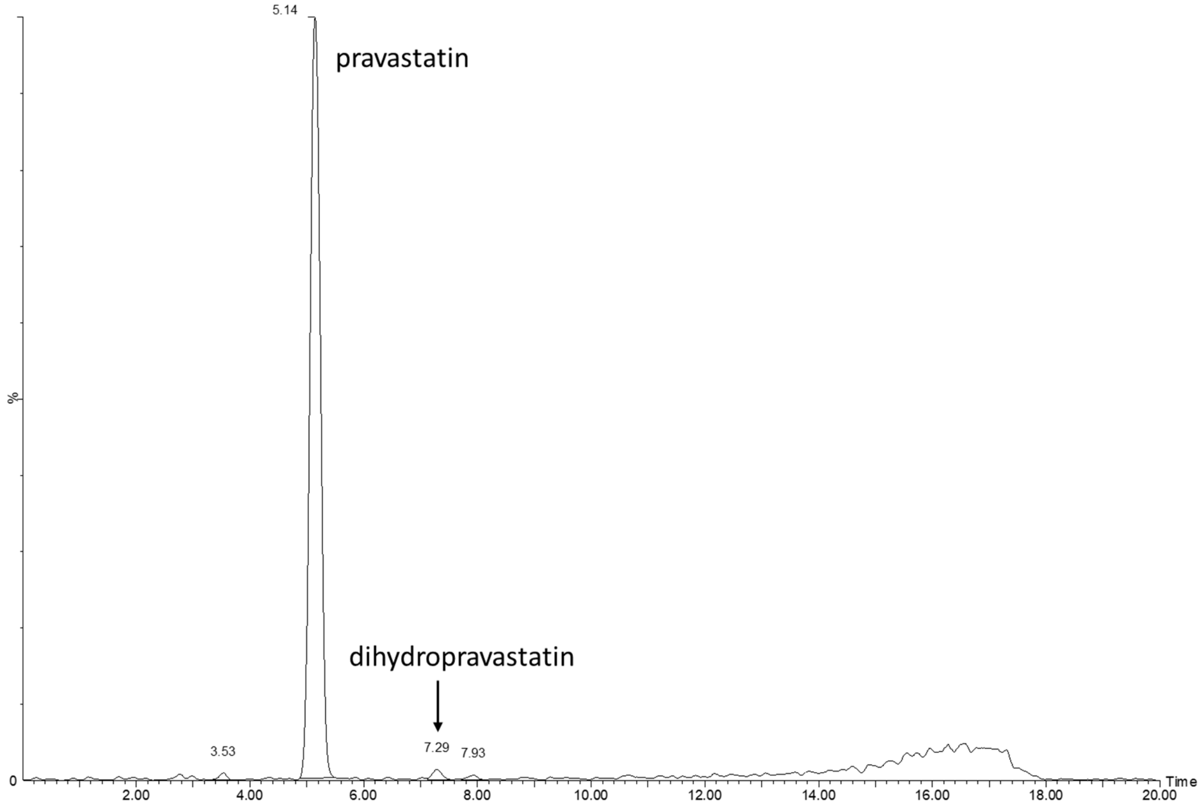

Supplementary Figure S2.: LC-MS selected ion chromatogram at m/z 465 of a partially purified pravastatin sample with pravastatin detected as the potassium adduct of the (M+2) isotope $[M+2+K]^+$ and dihydropravastatin as $[M+K]^+$

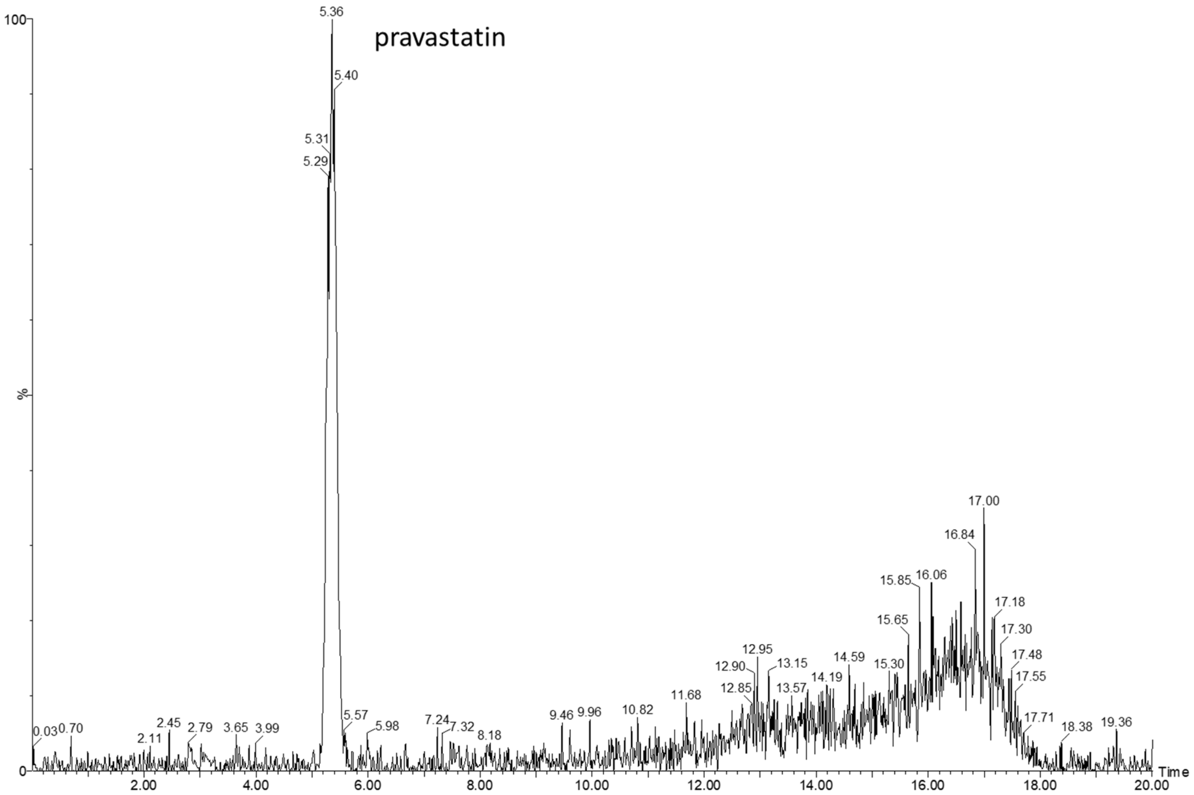

Supplementary Figure S3. LC-MS selected ion chromatogram at m/z 465 of the pravastatin API purified after recrystallization, with pravastatin detected as the potassium adduct of the (M+2) isotope $[M+2+K]^+$ and no dihydropravastatin

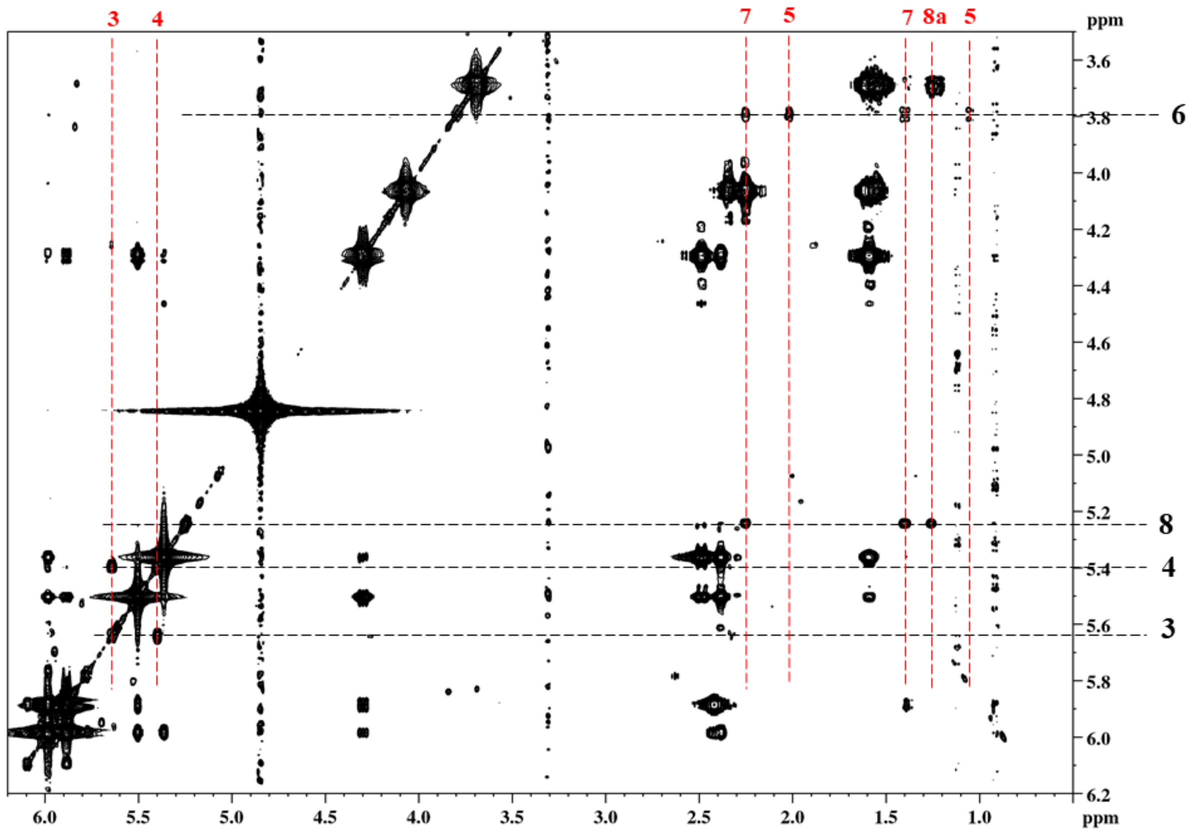

Supplementary Figure S4. COSY of pravastatin sodium salt (Sigma Aldrich) with connectivities of the 4a,5-dihydropravastatin impurity indicated with dashed lines.

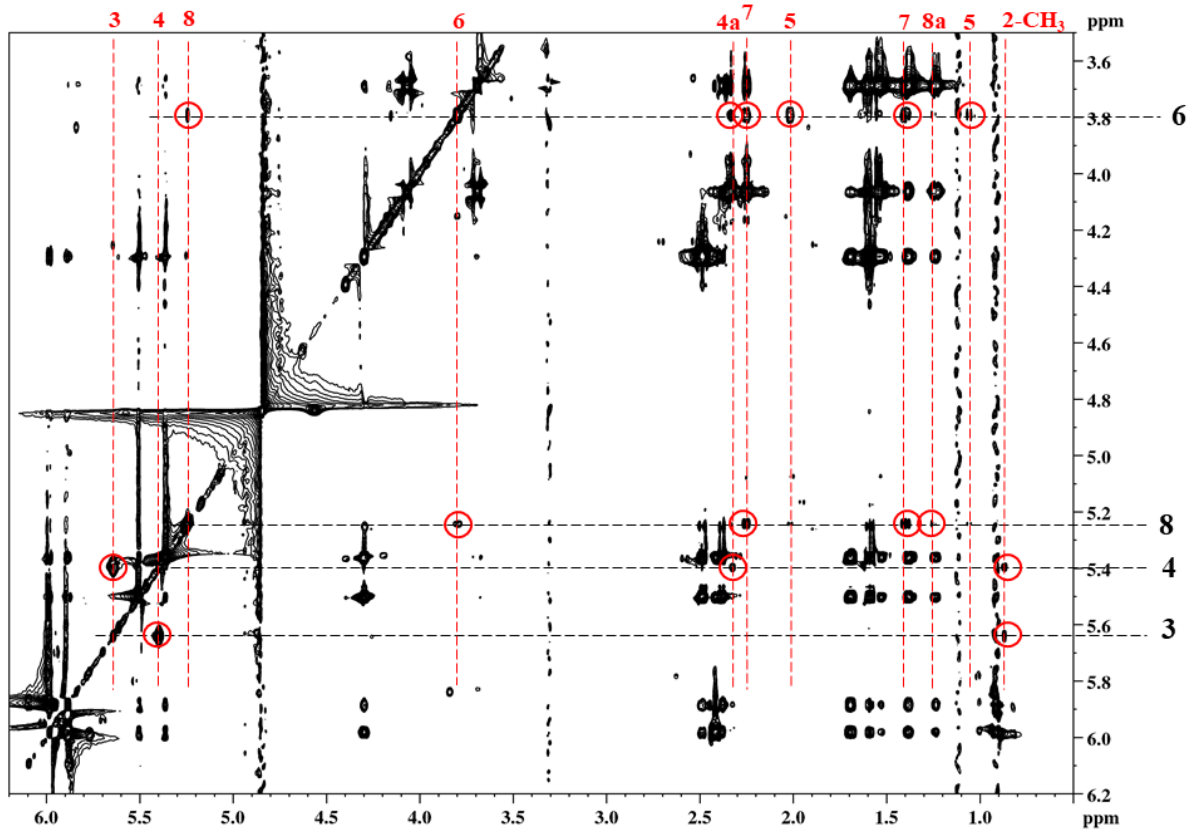

Supplementary Figure S5. TOCSY of pravastatin sodium salt (Sigma Aldrich) with connectivities of the 4a,5-dihydropravastatin impurity indicated with dashed lines.

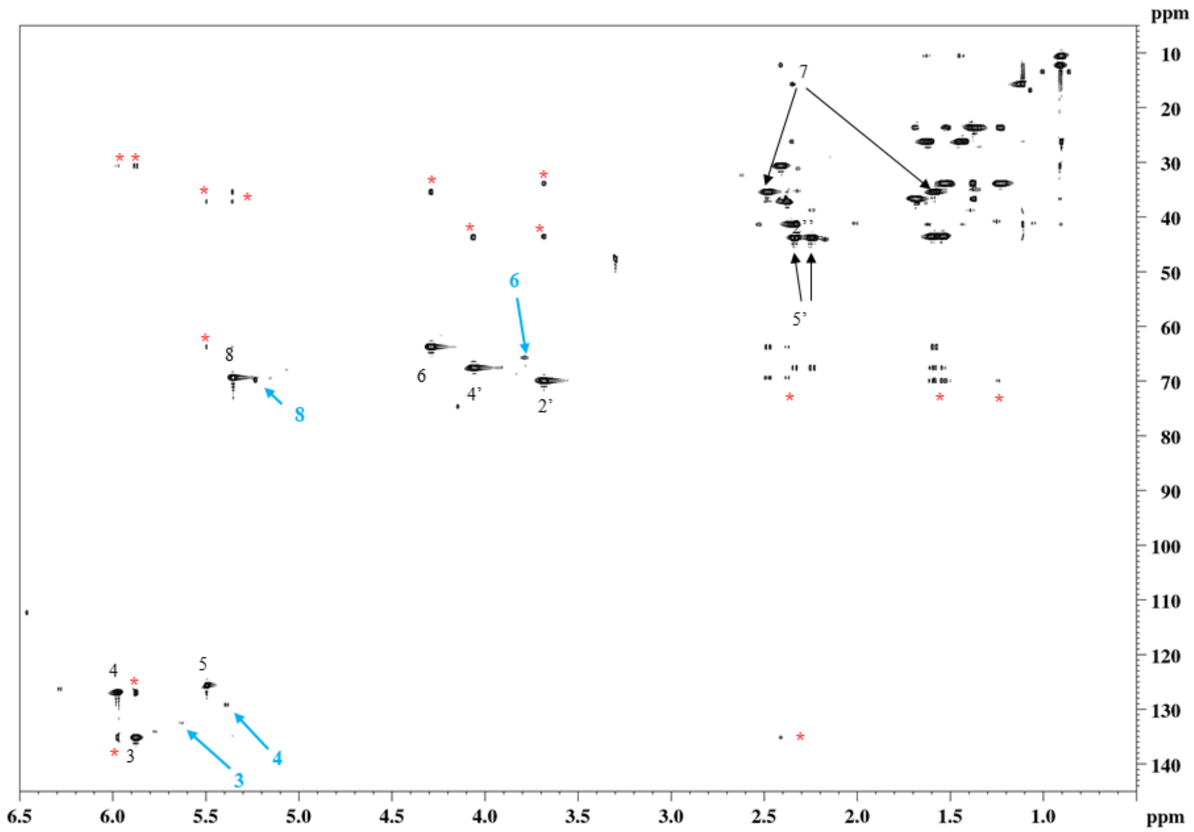

Supplementary Figure S6. HSQC of pravastatin sodium salt (Sigma Aldrich). Black numbers correspond to the assignment of pravastatin. The assignment of 4a,5-dihydropravastatin is indicated in blue, and the artefacts due to long-range couplings in pravastatin are indicated with red asterisks.

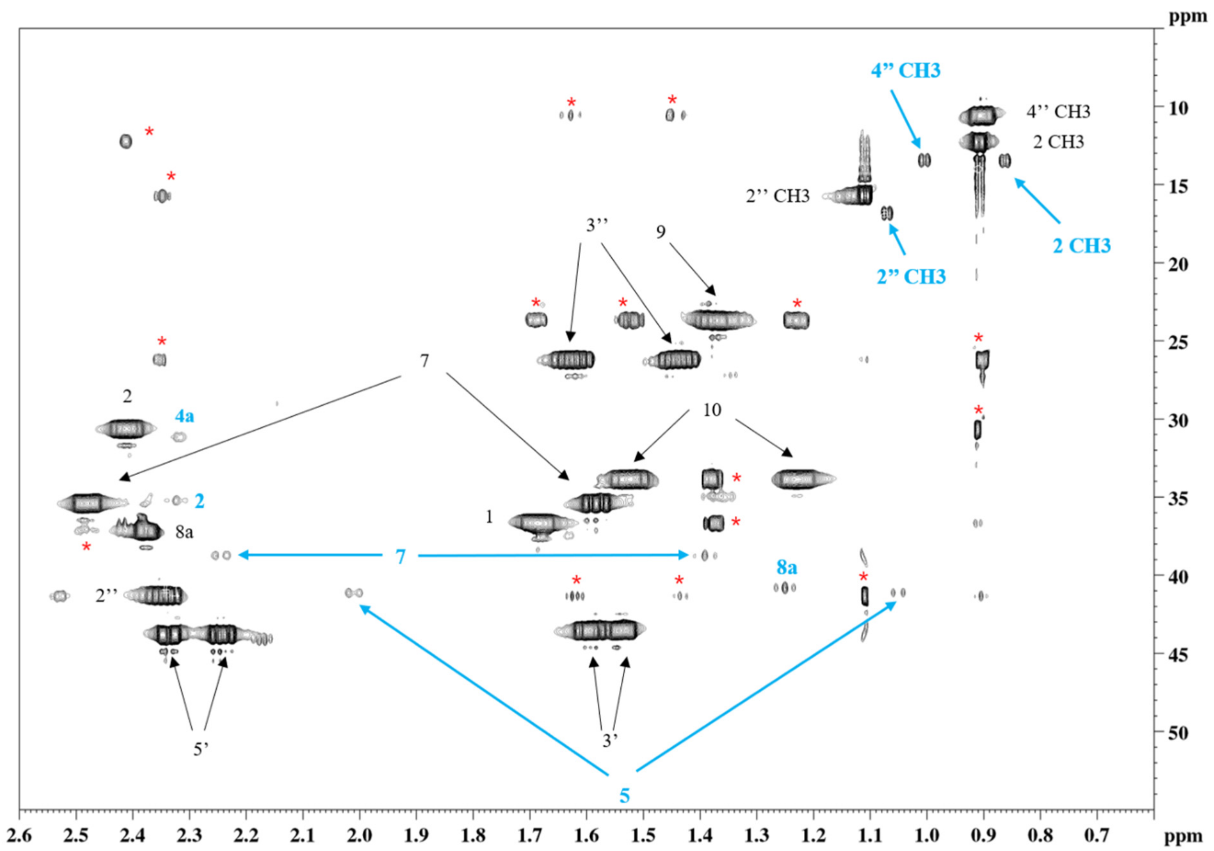

Supplementary Figure S7. High-field region of the HSQC of pravastatin sodium salt (Sigma Aldrich). Black numbers correspond to the assignment of pravastatin. The assignment of 4a,5-dihydropravastatin is indicated in blue, and the artefacts due to long-range couplings in pravastatin are indicated with red asterisks.

Supplementary Table S1. Chemical shifts of pravastatin and 4a,5 dihydropravastatin detected in methanol-d_4 at 300 K. 5 mg of pravastatin from Sigma Aldrich. Chemical shifts taken from HSQC spectrum. Chemical shift reference: methanol (δ ^{1}H = 3.300, δ ^{13}C = 47.67)

	Pravastatin		4a,5-dihydropravastatin	
Atom number	^{13}C	^{1}H	^{13}C	^{1}H
1	36.71	1.686	n.d	n.d.
2	30.60	2.408	35.23	2.323
3	135.14	5.877	132.54	5.634
4	126.92	5.975	129.18	5.389
4a	n.d.	n.a.	31.11	2.318
5	125.58	5.496	41.16	2.008 / 1.051
6	63.74	4.286	65.72	3.788
7	35.37	2.483 / 1.584	38.73	2.245 / 1.389
8	69.34	5.354	69.82	5.235
8a	37.17	2.378	40.79	1.250
9	23.64	1.377	n.d.	n.d.
10	33.76	1.521 / 1.226	n.d.	n.d.
CH_3(2)	12.20	0.907	13.38	0.862
2′	69.92	3.682	n.d.	n.d.
3′	43.50	1.587 / 1.541	n.d.	n.d.
4′	67.57	4.058	n.d.	n.d.
5′	43.84	2.338 / 2.243	44.90*	2.338 / 2.243*
2′′	41.31	2.345	n.d.	n.d.
3′′	26.15	1.626 / 1.439	27.15*	1.617 / 1.348*
4′′	10.44	0.901	n.d.	n.d.
CH_3(2′′)	15.59	1.109	n.d.	n.d.

n.d.: not detected, n.a.: not applicable, *: tentative assignment

The ^{13}C and ^{1}H NMR signals of the decalin ring system of 4a,5-dihydropravastatin were assigned unequivocally by means of COSY, TOCSY and HSQC spectra. Most of the signals of the two side chains could not be detected in 4a,5-dihydropravastatin due to the fact that these small signals of the 2% impurity are hidden by the strong signals of pravastatin. Because the difference between the main compound and the impurity is present in the decalin ring system, the signals of the latter part of the molecule differ sufficiently to enable the assignment of that part.

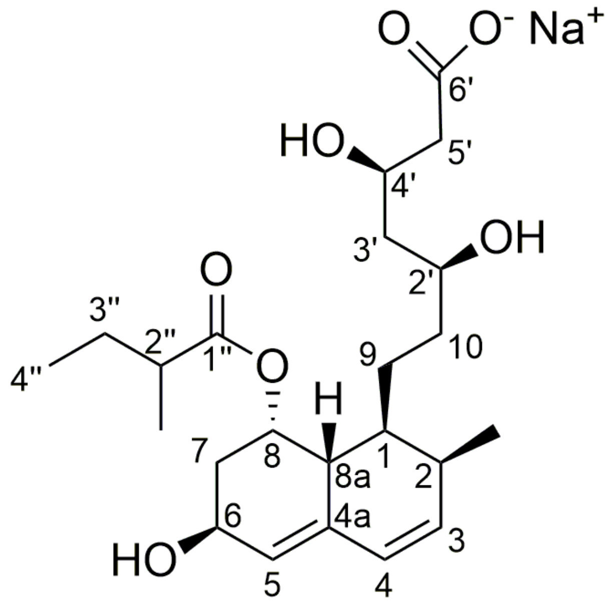

Supplement: Supplementary file 1 [file molecules-26-04685-s001.zip › molecules-1258737-supplementary.pdf]
